# Serum magnesium levels of children living with sickle cell anaemia at the Lagos University Teaching Hospital: a comparative cross-sectional study

**DOI:** 10.1186/s12887-025-06079-5

**Published:** 2025-10-22

**Authors:** Pauline Kasarachi Akowundu, Abideen Olurotimi Salako, Adeseye Michael Akinsete, Patricia Akintan

**Affiliations:** 1 Department of Paediatrics, Redeemers Health Village, Mowe, Redemption City, Ogun State Nigeria; 2 Nigerian Institute of Medical Research (NIMR), Yaba, Lagos State Nigeria; 3 College of Medicine, University of Lagos, Lagos, Lagos State Nigeria; 4 Department of Paediatrics, Lagos University Teaching Hospital, Lagos, Nigeria

**Keywords:** Sickle cell anaemia (SCA), Vaso-occlusive crises (VOC), Steady state. Serum Magnesium

## Abstract

**Background:**

Sickle cell anaemia (SCA) is a lifelong, chronic haemolytic disorder with episodic vaso-occlusive crises (VOC) and chronic end-organ damage. Red cell dehydration during a VOC changes permeability with loss of potassium and magnesium. Magnesium has been found to act via both Gardos and K-Cl channels, preventing red cell dehydration and reducing the propensity for sickling. This study assessed serum magnesium levels in children living with SCA in steady state and crisis, and the relationship between serum magnesium levels and severity of pain in VOC.

**Methods:**

A comparative cross-sectional study involving age- and gender-matched 180 children living with SCA aged 1–18 years (90 in VOC and steady state, respectively). The severity of pain was assessed using standard pain tools (FLACC and Wong-Baker FACES Pain scale), and a blood sample was taken for magnesium assay using the colorimetry method of analysis.

Categorical variables were presented as frequencies and percentages. Comparison of means between the two groups and according to magnesium status was carried out using an independent Student's t-test.

**Results:**

The mean age of participants in VOC and steady state was 8.49 ± 2.4 years and 8.62 ± 2.0 years, respectively. The mean serum magnesium of subjects in VOC was significantly higher compared to the steady state (0.85 ± 0.1 vs 0.83 ± 0.1 mmol/l, *p* = 0.032). The moderate to severe pain was the most common pain (63.3%), and the average serum magnesium was higher in the group with moderate to severe pain, but the difference was not statistically significant across the three groups (*p* = 0.315).

**Conclusion:**

VOC maybe associated with elevated serum magnesium in SCA.

## Introduction

Globally, sickle cell anaemia (SCA) is the most common haematologic disorder of public health importance [1]. The high prevalence of these non-communicable diseases, including sickle cell anaemia, contributes to the increased mortality of 36.4% in children below five years [2, 3].

Vaso-occlusive crises (VOC) remain the most typical clinical presentation of the disorder worldwide. It is a leading cause of emergency room visits and hospitalizations. VOC has devastating effects on the physical, psycho-social, financial, and quality of life of patients, caregivers, and society [4].

Magnesium (Mg) is the fourth most abundant cation in the body and acts as a vasodilator by increasing nitric oxide production, as well as improving endothelial function [5]. It is believed to be of benefit in SCA as it reduces inflammation and keeps the red cell hydrated, thereby reducing the frequency of VOC and improving the quality of life of the patient [6]. In individuals living with SCA, magnesium levels fall during a VOC due to abnormal red cell membrane permeability and dehydration [7, 8].

The reports on serum magnesium levels in individuals with sickle cell disease have been variable, ranging from low to normal [6, 9, 10]. In a study done by Oladipo *et al* [9] in Lagos, Nigeria, serum magnesium levels were found to be normal in both children with SCA in steady state and healthy controls (HbAA). In contrast, other similar studies have reported low levels of serum magnesium in children living with SCA, in Iraq, the USA, and Ghana [11–14].

The use of magnesium in the treatment of children and adults living with sickle cell anaemia reduced the length of hospital stay and duration of VOC [15, 16].

There is a dearth of studies done on HbSS children in steady state and VOC in Nigeria, and little attempt has been made to compare serum magnesium levels with the severity of pain in VOC. Most published studies on individuals with sickle cell anaemia have been conducted in adolescents and adults in steady state, without examining children in VOC. Additionally, few of the studies done in children were targeted at the late adolescent group, leaving out the majority of the younger age group [15, 17]. Despite the burden of the disease in Nigeria and attempts by the United Nations to enlighten the populace, SCA still poses a threat to global health. This study will fill the knowledge gap, improve the understanding and management of the disease, and help improve the care and outcome of affected children.

## Methods

The study was a comparative, cross-sectional study carried out over twelve months, from July 2020 to June 2021. The purposive sampling method was used. The study participants were recruited from the Pediatric Haematology Clinic, Children's Emergency Room, and those admitted into the Paediatric ward of the Lagos University Teaching Hospital. The study participants included children with SCA in VOC, which served as the subjects. At the same time, the control group consisted of children in steady state, age and gender-matched, who met the inclusion criteria and were recruited after informed consent was obtained. For both study groups, the genotype was confirmed using the hemoglobin electrophoresis method.

The inclusion criteria used to select those in steady state include: Children diagnosed with sickle cell anaemia aged 1–18 years in a constant state. [A steady state is defined as being free from pain, infection, or other acute illness for three months and not having a blood transfusion for a period of three 3 months before the study] [2]. And also those whose parents or guardians gave consent, and children aged 7 years and above who gave assent. Those in VOC who gave assent/consent also partook in the study. Children on magnesium-containing supplements, confirmed chronic diseases (heart, kidney, digestive), those who did not give assent/consent, and did not meet the inclusion criteria were all excluded from this study.

The sample size was determined using the formula for the comparison of two means [18].

$$\text{N}=2\left(\frac{\left(\text{Z}\mathrm\alpha+\text{Z}\mathrm\beta\right)\text{s}}{\text{d}}\right)^2$$

Where,

N –sample size

Zα –standard normal deviate at 5% level of significance = 1.96

Zβ –standard normal deviate at β probability, which at 80% power is 0.84

S- sample standard deviation from a similar local study [19]. = 0.12

d- tolerable margin of error = 0.05

$${2\left(\frac{\left(1.96+0.84\right)0.12}{0.05}\right)}^2=90$$

This consisted of 90 children in vaso-occlusive crises and 90 who were in a steady state. A total of 180 participants were recruited for this study.

Ethical approval was obtained from the Health Research and Ethics Committee [HREC] of the Lagos University Teaching Hospital, with amendment of the study granted before the commencement of the study.

The interviewer administered a pretested questionnaire to obtain biodata, relevant medical history (chronic renal, cardiovascular, or digestive illness), after which a detailed clinical examination was carried out. Blood specimen was taken for serum magnesium in a lithium heparin sample bottle. For participants in this study, a documented record of their genotype was confirmed from the clinic records. The severity of pain in subjects with VOC was assessed using the FLACC (Face, Leg, Activity Cry, and Consolability) pain scale for children 1–7 years and the Wong and Baker FACES pain scale for children above 7 years. These two pain score charts have previously been validated in African children [20, 21].

There are several methods commonly employed for the measurement of serum magnesium: atomic absorption spectroscopy, colorimetric assay, and fluorimetric measurement [22, 23]. However, the simple and rapid colorimetry test was used in this study. This method was preferred because it has very high accuracy, good precision, and is time-efficient.

Blood sample was centrifuged at 1600 relative centrifugal force (rcf) for 5 min using the Appendorf centrifuge 5702 machine. The sample analysis was done using the Roche Cobas c 311 analyzing machine and the MG-2 kit (magnesium gen 2). These samples were located by a probe, which transferred them into the reaction cell. The reagent, TRIS (tris(hydroxymethyl) aminomethane/6-aminocaproic acid buffer), was then added, and the sample was incubated at 37 °C for approximately 5 min. Following this, the second reagent, xylidyl blue, was added, and the reaction cell was then passed through the photometer lamp and absorbance read at 505/600 nm wavelength. Magnesium was measured photometrically via a reduction in xylidyl blue absorbance. In an alkaline solution, magnesium forms a purple complex with xylidyl blue. Serum magnesium results were recorded in mmol/l. The serum levels were categorized as normal at: 0.65–1.05 mmol/l or (1.5—2.3 mg/dl) or (1.2–1.9 mEq/L) [24]. Hypomagnesemia = < 0.65 mmol/l (< 1.5 mg/dl) and Hypermagnesemia = > 1.05 mmol/l (> 2.3 mg/dl).

Data collected was analysed with a statistical package for social sciences version 26 [SPSS Inc., Chicago, IL]. Normal distribution was determined using the Kolmogorov–Smirnov test. Normally distributed numerical data (magnesium level) was presented as means and standard deviations. In contrast, skewed data (duration of pain) was presented using the median and interquartile range. Comparison of means between the two groups (VOC and steady state) and according to magnesium status (Normal and elevated) was carried out using an independent Student's t-test. Association between categorical variables was carried out using the chi-square test and Fisher's exact test. Categorical variables (serum magnesium) are presented as frequencies and percentages. A p-value of less than 0.05 was regarded as statistically significant.

## Results

### Socio-demographic characteristics of study participants

A total of one hundred and eighty (180) children were enrolled in the study: 90 subjects in each of the two groups, vaso-occlusive crises (subjects) and steady state (controls).

The mean age of the participants at enrollment into the study was 8.49 ± 2.4 years and 8.62 ± 2.0 years for those in VOC and steady state, respectively. There was a slight male preponderance, with a male: female ratio of 1.05:1, respectively, in the whole study.

There were no significant differences in demographic characteristics between the two groups, as shown in Table 1.

**Table 1:** Socio-demographic characteristics of study participants

	**VOC (*n* = 90)** **n (%)**	**Steady state (*n* = 90)** **n (%)**	**Total**	**χ**^**2**^	***p*-value**
Age group (Years)					
1–6	30 (33.3)	30 (33.3)	60 (33.3)	0.000	1.000
7–12	46 (51.1)	46 (51.1)	92 (51.1)		
13–18	14 (15.6)	14 (15.6)	28 (15.6)		
Mean ± SD	8.49 ± 2.4	8.62 ± 2.0		0.341*	0.891
Gender					
Male	46 (51.1)	46 (51.1)	92 (51.1)	0.000	1.000
Female	44 (48.9)	44 (48.9)	88 (48.9)		
Social class					
Upper	6 (6.7)	2 (2.2)	8 (4.5)	3.988	0.136
Middle	25 (27.7)	35 (38.9)	60 (33.3)		
Lower	59 (65.6)	53 (58.9)	112 (62.2)		

^*^Independent student t-test

### Comparison of serum magnesium levels in VOC and steady state

Table 2 compares serum magnesium among children with SCA in VOC and steady state, revealing significantly higher mean levels in VOC (0.85 ± 0.1) than in steady state (0.83 ± 0.1, *p* = 0.032), with both elevated and deficient magnesium levels observed in 4(4.4%) of VOC subjects, compared to 1(1.1%) for each in steady state.

**Table 2:** Comparison of serum magnesium levels in VOC and steady state

	**VOC** **(*n* = 90)** **n (%)**	**Steady state** **(*n* = 90)** **n (%)**	**Total**	**χ**^**2**^	***p*-value**
Deficient	4 (4.4)	1 (1.1)	5 (2.8)	3.812	0.149
Normal	82 (91.1)	88 (97.8)	170 (94.4)		
Elevated	4 (4.4)	1 (1.1)	5 (2.8)		
Mean ± SD	0.85 ± 0.1	0.83 ± 0.1		1.748*	0.032**

^*^Independent student t test

^**^p < 0.05- significant p value

### Severity of pain among children with SCA in VOC

**Fig. 1:**
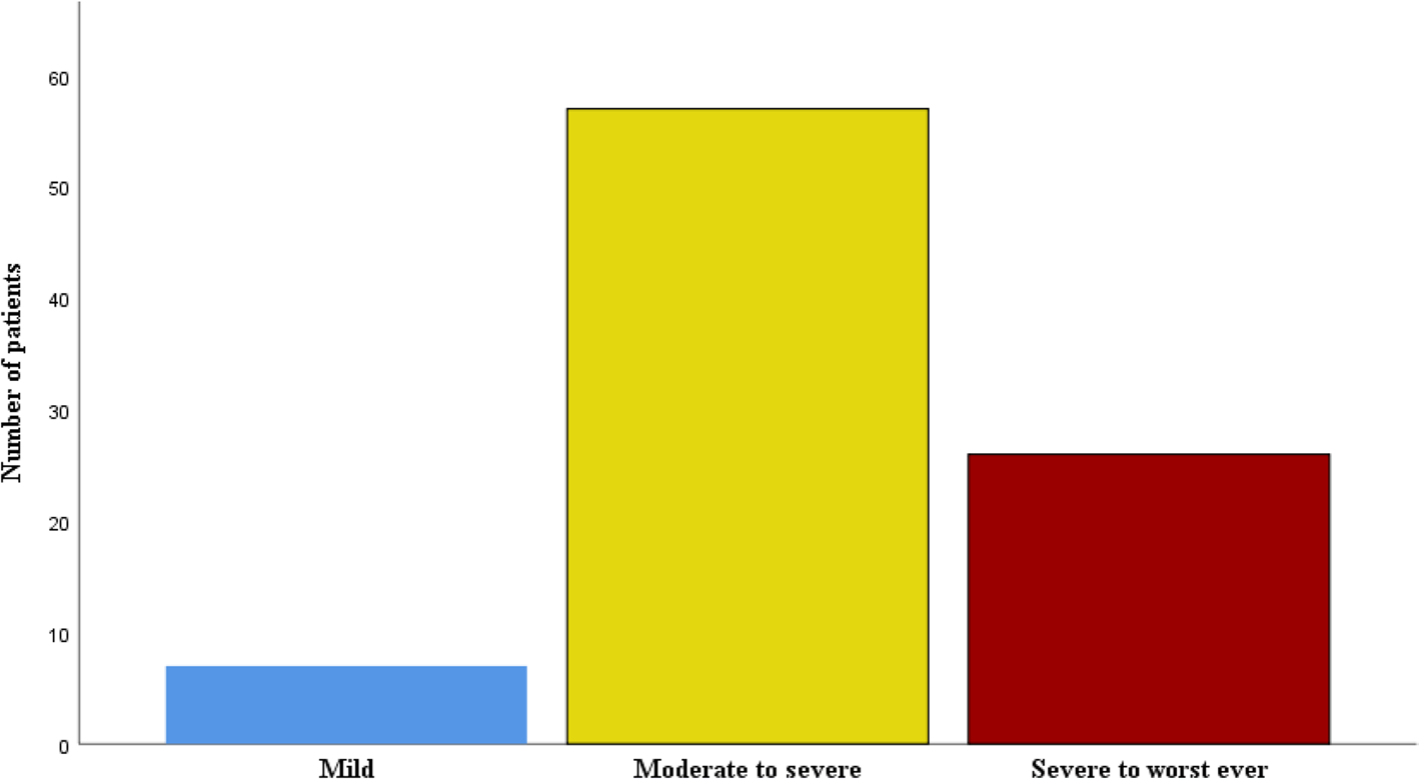
Severity of pain among children with SCA in VOC. Mild pain score: 1–3, Moderate—Severe pain score: 4–6, Very Severe –Worst pain ever: 7–10

The moderate to severe pain was the most common pain, 57 (63.3%), while the mild pain category, 7 (7.8%), was the least common among children in VOC, as seen in Fig. 1

### Association between mean serum magnesium level and severity of pain in children with SCA in VOC

The relationship between pain severity in VOC and serum magnesium levels is shown in Table 3. There was no statistically significant difference between the mean magnesium level across the three groups (*p* = 0.315). No significant association between severity of pain and magnesium level (*p* = 0.820).

**Table 3:** Association between mean serum magnesium level and severity of pain in children with SCA in VOC

	**Mean ± SD**	***p*-value**	**Deficient (*n* = 4)**	**Normal (*n* = 82)**	**Elevated (*n* = 4)**	***p*-value**
Pain Level						
Mild	0.80 ± 0.1	0.315^#^	0 (0.0)	7 (100.0)	0 (0.0)	0.820^*^
Moderate -severe	0.87 ± 0.1		2 (3.5)	52 (91.2)	3 (5.3)	
Very severe- worst pain	0.84 ± 0.1		2 (7.7)	23 (88.5)	1 (3.8)	

^#^Analysis of variance

^*^Chi-square

## Discussion

In this study, most (97.8%) of the children in steady state had a normal level of serum magnesium. This finding is in keeping with a study by Oladipo et al [9] in Lagos, Nigeria, who reported a 100% prevalence of normal serum magnesium. Only 1.1% of children in the index study had low serum magnesium. However, contrary to the current findings, a low mean serum magnesium level in VOC and states was reported by other studies [10, 11, 17]. The variations in the serum magnesium levels among children in steady state can be explained by genetic, dietary, environmental, and geographic variations among study subjects [11]. Furthermore, different methods of serum magnesium measurement used in the various studies can also be implicated, as the affordable and more common colorimetry method, which has gained widespread use in developing countries, was used by both the index study and Oladipo et al. [9]. The other plausible explanation for the low serum magnesium includes increased membrane permeability and magnesium efflux via increased activity of the sodium/magnesium exchanger, glomerular hyperfiltration, and excessive urinary excretion of many trace elements due to damaged glomeruli and loss of trace element carrier proteins [11].

Among children in vaso-occlusive crisis, 4.4% in the index study were found to have low levels of serum magnesium. Additionally, these values vary widely, with a prevalence of 35% reported by Garba et al [19] in Zaria, 50% by Okocha et al [14] in Anambra, Nigeria. Similarly, 4.4% of those in VOC had elevated levels of serum magnesium. The elevated levels of serum magnesium could have occurred because during a VOC, there is a further increase in the resting energy expenditure in a background of hyper-catabolic state, leading to an elevated red cell hemolysis and with an exaggerated inflammatory response during a crisis, which would result in release of intracellular magnesium to the extracellular space [9, 25]. Furthermore, the presence of a VOC could result in increased red cell permeability and dehydration, with concurrent loss of potassium, chloride, and water through connected pathways, especially the Gardos Channel, and a release of magnesium into the extracellular space [7, 8]. Hence, the serum magnesium value in the acute painful crises subjects might not be an accurate picture of their serum magnesium levels, considering that there is an increased intracellular release of magnesium into the plasma/extracellular space during crises, and baseline levels of serum magnesium in these subjects were not evaluated before the crises. The majority of the subjects in this study in VOC (91.1%) and in steady state (97.8%) had normal levels of serum magnesium. This could be alluded to the fact that most of our regular local diet is rich in magnesium, and such foods are cheap and readily available [9]. There was also a higher prevalence of normal serum magnesium in steady state than in VOC, and possible reasons for this finding include underlying ill health, redistribution of nutrients, increased energy expenditure, and nutrient demand by the body during a VOC [25–27].

There was no association between serum magnesium and the severity of pain in vaso-occlusive crises (VOC) in this study. This lack of association may be a consequence of normal levels of serum magnesium creating an analgesic effect, in the presence of factors that can precipitate painful crises (extremes of weather, strenuous activities) [28]. Furthermore, individual pain perception/sensitivity or threshold, ethnocultural beliefs about pain, including a biased/subjective scoring, would have contributed to the finding in the index study. The genetic makeup of the children, including psychosocial support, emotional/mood, behavioral, and lifestyle factors, may interact to have an impact on pain tolerability, thereby contributing to this finding. Additionally, personal factors like individual pain coping strategies, spiritual beliefs, and attempts to create distractions from pain could have led to the lack of association [29]. The use of analgesics (morphine, ibuprofen) by subjects during a VOC may have also played a role in modifying the severity of pain. These analgesics have also been found to alter levels of serum magnesium by either reducing excretion or increasing its absorption, and this may have accounted for the report in this study.

It is important to note that during a vaso-occlusive crisis (VOC), the horde of events will result in a release of inflammatory substances, including substance P, which facilitates transmission of pain via the NMDA receptor, and also the release of intracellular magnesium via activation of red cell membrane channels [7, 8, 30]. It is a fact that magnesium blocks the N-Methyl-D-Aspartate receptor (NMDA), resulting in an analgesic effect, and this analgesic effect is related to the prevention of central sensitization caused by inflammation and tissue damage that occurs during a VOC [31]. With the exaggerated release of intracellular magnesium during VOC, activities of the K-Cl and Gardos channels are impaired, thereby preventing red cell dehydration and sickling. In addition, the release of magnesium in crises may contribute to vasodilation through endothelium-dependent release of nitric oxide (NO) and inhibition of calcium in smooth muscle, which in turn reduces VOC. The above factors could have led to the prevalence of moderate to severe pain, as well as the lack of association between magnesium and pain in this study. There is a paucity of studies done on the relationship between serum magnesium and severity of pain in VOC; hence, larger longitudinal studies among children living with SCA may be needed to determine any association between pain and serum magnesium. Future research should focus on investigating the clinical outcomes of nutritional deficiencies in patients with SCD, as well as exploring the potential benefits of implementing nutritional supplement programs to mitigate the disease's adverse effects on the human body.

## Limitations

The cross-sectional nature of the study limited the assessment of magnesium in the same subjects before and during crises. Additionally, the potential use of unorthodox supplements containing magnesium could not be determined among the study subjects.

## Conclusion

Serum magnesium levels were found to be significantly elevated in children living with sickle cell anemia in vaso-occlusive crises (VOC) compared to those in a steady state.

There was no relationship between the level of serum magnesium and the severity of pain in VOC. Vaso-occlusive crises maybe associated with elevated serum magnesium.

## Data Availability

All data generated and analyzed during the current study are included in this published article.

## Abbreviations

HbAA: Hemoglobin AA
SCA: Sickle cell anaemia
VOC: Vaso-occlusive crisis
HbSS: Hemoglobin SS genotype
